# Imaging and Quantification of Myelin Integrity After Injury With Spectral Confocal Reflectance Microscopy

**DOI:** 10.3389/fnmol.2019.00275

**Published:** 2019-11-19

**Authors:** David G. Gonsalvez, SangWon Yoo, Jessica L. Fletcher, Rhiannon J. Wood, Georgina A. Craig, Simon S. Murray, Junhua Xiao

**Affiliations:** Neurotrophin and Myelin Laboratory, Department of Anatomy and Neuroscience, School of Biomedical Sciences, Faculty of Medicine, Dentistry and Health Sciences, The University of Melbourne, Parkville, VIC, Australia

**Keywords:** imaging, myelination, demyelination and remyelination, myelin debris, cuprizone model

## Abstract

Developing a high-throughput approach to quantify the extent of myelin integrity in preclinical models of demyelinating diseases will enhance our capacity to identify novel therapies for myelin repair. In light of the technical limitations of electron microscopy and immunohistochemical analyses of myelination, we have utilized a novel imaging technique, spectral confocal reflectance (SCoRe) microscopy. SCoRe takes advantage of the optically reflective properties of compact myelin, allowing the integrity of compact myelin to be quantified over the course of the cuprizone-induced model of central demyelination. We applied SCoRe imaging on fixed frozen brain sections. SCoRe analysis of control mice identified an increase in corpus callosum myelination during the period of cuprizone administration and recovery, suggesting that the normal developmental processes of myelination are ongoing at this time. Importantly, analysis of mice subjected to cuprizone identified a significant reduction in compact myelin in both rostral and caudal corpus callosum compared to age-matched control mice. SCoRe microscopy also allowed the visualization and quantification of the amount of myelin debris in demyelinating lesions. Combining SCoRe imaging with immunohistochemistry, we quantified the amount of myelin debris within IBA-1+ microglia and found that 11% of myelin debris colocalized in microglia irrespective of the callosal regions, with the vast majority of debris outside of microglia. In summary, we have demonstrated that SCoRe microscopy is an effective and powerful tool to perform both quantitative and qualitative analyses of compact myelin integrity in health or after injury *in vivo*, demonstrating its future application in high-throughput assessments and screening of the therapeutic efficacy of myelin repair therapies in preclinical animal models of demyelinating diseases.

## Introduction

Myelin is a specialized protein–lipid bilayer sheath that extends from myelin-producing glia, the oligodendrocytes in the central nervous system (CNS), and Schwann cells in the peripheral nervous system. Myelin sheaths surround axons, forming a compact multilamellar structure, which not only facilitates rapid conduction of electrical signals along axons but also provides metabolic and trophic support to neurons (Kramer-Albers et al., 2007; Nave, 2010). Adequate generation and maintenance of compact myelin are critical for the normal function of the nervous system, and any disruptions in the process of myelination or damage to myelin can result in demyelination and subsequent neuronal degeneration or loss. Most notably, this occurs in multiple sclerosis (MS), where there is a direct autoimmune attack against the myelin sheath. During myelin damage, the fragmented myelin sheaths aggregate and form large, irregular clusters of myelin debris (Prineas et al., 1984). Efficient removal of myelin debris by microglia is known to be a prerequisite for facilitating myelin repair (Kotter et al., 2005; Lampron et al., 2015). Therefore, techniques that enable quantitative and high-throughput analyses of changes to compact myelin, including the visualization of myelin debris in diseases such as MS or its preclinical animal models, are highly desirable for the development of new myelin repair therapies. However, current imaging techniques of studying compact myelin integrity *in vivo* have clear limitations.

Conventionally, transmission electron microscopy (TEM) has been the only method able to produce a satisfactory resolution of myelin ultrastructure and debris. Despite the unparalleled resolution in EM, the technique has major downsides in terms of the extensive tissue preparation process and sampling capacity (Skripuletz et al., 2011). Immunohistochemical staining against myelin protein is a common approach used as a surrogate measure for the extent of myelination. However, in immunostaining, in order to expose the proteins “hidden” within the tightly compacted myelin sheaths, antigen retrieval procedures and detergents are employed to disrupt the structure of the lipid-rich myelin membranes, inevitably altering the level of myelin proteins exposed for antibody detection. This represents a confounding factor for immunostaining-based analysis of myelination. Recently, a novel label-free reflectance imaging technique that allows direct visualization of myelin was developed (Schain et al., 2014). The technique, known as spectral confocal reflectance (SCoRe) microscopy, exploits a unique feature of compact myelin, which optically reflects incident laser lights (Schain et al., 2014). Being label-free and utilizing conventional confocal systems, SCoRe microscopy involves minimal tissue preparation and allows sampling of a substantially greater area of the CNS (Schain et al., 2014; Hill et al., 2018; Hughes et al., 2018). However, it is unclear if SCoRe microscopy enables quantitative analysis of the extent of myelin damage and repair in animal models of CNS demyelination.

In this study, we used SCoRe microscopy to quantify changes to compact myelin and myelin debris in the cuprizone-induced murine model of CNS demyelination. Here, we show that SCoRe imaging is able to detect a significant difference in the extent of compact myelin between cuprizone-challenged mice and age-matched healthy control groups, in rostral and caudal corpus callosum. In the cuprizone-challenged animals, we detected and quantified the presence of atypical reflection of myelin (myelin debris), most of which persists outside macrophages even after 1 week of remyelination. Together, results of this study demonstrates that SCoRe is a highly reproducible and powerful technique that allows quantification of compact myelin integrity including myelin debris *in vivo*.

## Materials and Methods

### Animals and Cuprizone Administration

**FIGURE 1:**
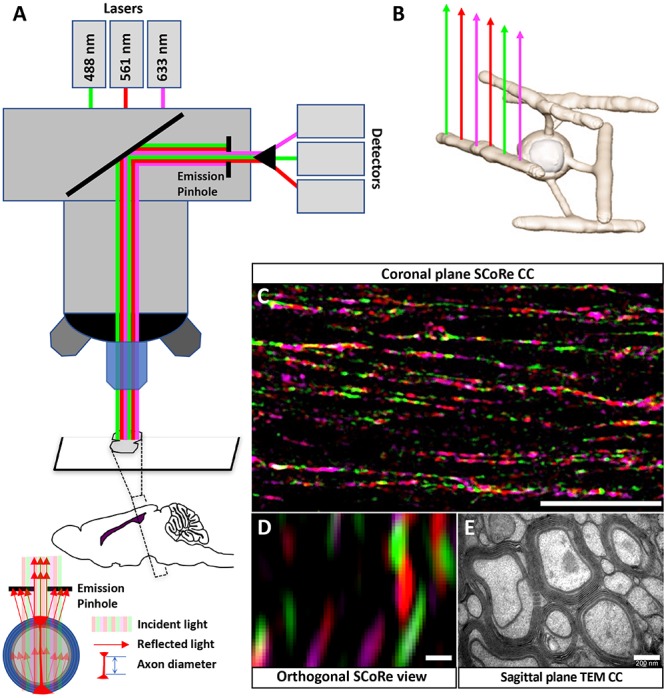
Using SCoRe to visualize compact myelin in fixed frozen brain sections. **(A)** Schematic representation of microscope configuration and the reflected signal captured in SCoRe. The picture of the reflection pattern indicates how SCoRe imaging can be utilized to estimate axon diameter. **(B)** Different wavelengths of light are preferentially reflected back along the length of an internode, which has been attributed to changes in thin film interference along the length of an internode (Schain et al., 2014). **(C)** Single optical slice of a coronal section through the corpus callosum demonstrating how different wavelengths of light are reflected along the myelin internode (scale bar, 10 μm). **(D)** Orthogonal slice from a *Z*-stack SCoRe image of callosal axons (scale bar, 200 nm). **(E)** TEM section taken through the corpus callosum indicating individual myelin lamellae compacted around axons (scale bar, 200 nm). Panels **(D,E)** are taken from the same animal, where half of the brain went to TEM processing and the other half went to SCoRe imaging. The SCoRe reflection signal is stereotypical for healthy myelin. SCoRe, spectral confocal reflectance; TEM, transmission electron microscopy.

C56BL/6 female mice were used in all experiments. Mice were housed in specific pathogen-free conditions at the Melbourne Brain Centre Animal Facility. All animal procedures were approved by The Florey Institute of Neuroscience and Mental Health Animal Ethics Committee and followed the Australian Code of Practice for the Care and Use of Animals for Scientific Purposes. Mice were housed three animals per cage. A minimum of 1 week of acclimation time to the new environment was allowed prior to the start of cuprizone administration. Demyelination was induced by administration of manufactured rodent chow pellet containing 0.2% cuprizone [bis(cyclohexanone)oxaldihydrazone; Envigo RMS Division, Indianapolis, IN, United States] to 6- to 8-week-old C57BL/6 mice for 6 weeks (Figure 1). Age-matched control mice were kept on normal chow formulated to have the same nutrient levels as the cuprizone pellets. Food pellets and cages were changed twice a week. Water was provided *ad libitum*.

### Tissue Collection

**FIGURE 2:**
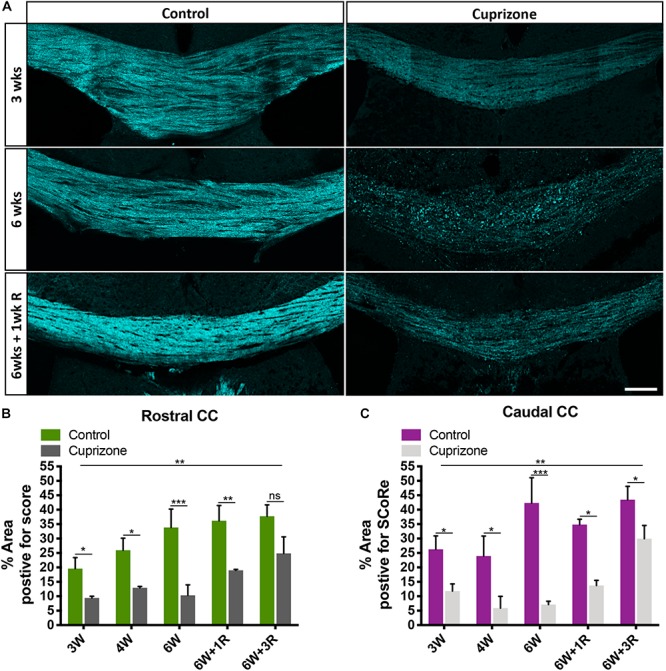
SCoRe imaging can be used to quantify changes to compact myelin after *in vivo* myelin injury. **(A)** Representative SCoRe images of myelin signals in midline corpus callosum demonstrating changes to compact myelin in cuprizone-challenged mice compared with the age-matched healthy control (scale bar, 150 μm). **(B,C)** Quantification of the myelinated area positive for SCoRe signal (pixels) as a percentage of total area measured. The SCoRe signal is significantly reduced in both rostral **(B)** and caudal **(C)** corpus callosum of all cuprizone-challenged mice compared with age-matched healthy control, indicative of demyelination. For each data, point *n* = 3 mice per group, statistics stars indicate a significant interaction (large line) between time and cuprizone exposure determined by two-way ANOVA, multiple comparisons, and Tukey’s *post hoc* testing (short lines to indicate pairwise comparisons) (^∗^*P* < 0.05, ^∗∗^*P* < 0.01, ^∗∗∗^*P* < 0.001; data = mean ± SD). SCoRe, spectral confocal reflectance.

All mice were anesthetized using isoflurane and were intraperitoneally injected with sodium pentobarbital (80 mg/kg). Mice were then fixed via transcardial perfusion using 0.1 M of sterile phosphate-buffered saline (PBS) followed by 4.0% paraformaldehyde (PFA) in 0.1 M of PBS at a rate of 4 ml/min using an NE-1000 programmable single syringe pump. Brains were dissected out and post-fixed overnight at 4°C in 4% PFA. Tissue was then washed in PBS and placed in 30% sucrose overnight at 4°C; the brains were next embedded in optimal cutting temperature (OCT) and frozen in isopentane cooled with dry ice. Serial sections in a coronal orientation were captured on a cryostat microtome at 25-μm thickness (Figure 2A).

### Immunohistochemistry

For immunostaining without membrane permeabilization, 25-μm-thick brain sections were incubated with primary antibodies (see Table 1) prepared in a diluent containing PBS with 10% normal donkey serum (NDS) for at least 3 days at room temperature. Sections were then incubated with secondary antibodies prepared in PBS for 2 h at room temperature in the dark. Hoechst 33342 (Sigma, 23491-52-3) was added to secondary antibodies as a nuclear marker. For some myelin basic protein (MBP) immunostaining with membrane permeabilization steps, slides with 25-μm-thick brain sections were dipped in cold methanol (80%) for 10 min followed by washes in PBS for three times. Sections were then incubated with primary antibodies prepared in a diluent containing PBS with 10% NDS and 0.3% Triton X-100 for at least 3 days, followed by the incubation with appropriate secondary antibodies for a minimum of 2 h.

**TABLE 1:** List of primary and secondary antisera.

	**Source**	**Concentration**
**Primary antisera**		
Rat anti-myelin basic protein	Millipore, MAB386	1:200
Rabbit anti-IBA-1	WAKO 019-19741	1:1000
Goat anti-IBA-1	Abcam (ab5067)	1:200
Secondary antisera and reagents		
Alexa 647 donkey anti-mouse	Abcam A31571	1:200
Alexa 594 donkey anti-rat	Abcam A21209	1:200
Alexa 488 donkey anti-rabbit	Abcam A21206	1:200
Alexa 488 donkey anti-goat	Abcam A15077	1:200
Hoechst 33342 (bisbenzimide)	Sigma 23491-52-3	1 μg/ml

### Spectral Confocal Reflectance Microscopy and Image Analysis

All images were captured on a Zeiss LSM 880 (Airyscan) or Zeiss LSM780 confocal microscope using a water immersion objective (Zeiss W Plan-Apochromat 40/1.0 NA DIC or Zeiss W Plan-Apochromat 20/1.0 NA DIC) using 458-, 561-, and 633-nm laser wavelengths passed through the acousto-optical tunable filters (AOTF) 488–640 filter/splitter and a 20/80 partially reflective mirror. The reflected light was collected using three photodetectors set to collect light through narrow bands defined by prism and mirror sliders, centered around the laser wavelengths of 488, 561, and 633 nm (Figure 1A). To perform quantitative analysis, the channels from each photodetector were overlaid as one composite image. Compact myelin was assessed by positive pixel identification using a minimum threshold cutoff in FIJI (v1.52k National Institutes of Health, United States). To generate a percentage, the area positive for SCoRe signal was divided by the total area of the region of interest (ROI). In all cases, the ROI was a trace made to include the total cross-sectional area of the corpus callosum in each section analyzed (Govier-Cole et al., 2019).

Normal-appearing compact myelin reflects light in a very stereotypical pattern (Schain et al., 2014; Hill et al., 2018). Only reflected light that passes through a pinhole can be identified by the detector, and internodes have a stereotypical appearance of long thin lines. Compacted myelin debris is present as round and large aggregates, which are strikingly distinct from the linear appearance of normal compact myelin. This compacted debris/demyelination can be identified using semiautomated process: The threshold function in FIJI was used to create a binary mask of the combined SCoRe signal and the “Analyze Particles” tool was then applied to identify any SCoRe signal that had high (0.75–1.0) circularity with a diameter of 0.6 μm or larger. These parameters were chosen as reflected signal from undamaged myelin has a very stereotypical oblong appearance, and although cortical axons can exceed 0.6 μm in diameter, the reflected width of an internodal segment is rarely greater than 0.5 μm. All selections were inspected by an observer blind to sample identity to ensure no erroneous detection of healthy compact myelin occurred. To identify debris colocalized within IBA-1-positive macrophages, first, a binary mask on the IBA-1 immunosignal was created, and then an analysis of particle criteria was applied to exclude normal myelin and positively quantify area of pixels positive for atypically reflected myelin within microglia. All volumetric reconstructions of IBA-1 cells or SCoRe were carried out in IMARIS software. All analyses were performed blinded to sample identity.

### Transmission Election Microscopy

To prepare sections for TEM, the whole brain fixed in 4% PFA overnight was cut through the sagittal midline, with the first millimeter of both hemispheres transferred to Karnovsky’s buffer overnight and washed in 0.1 M of sodium cacodylate, before being embedded in epoxy resin at the Peter MacCallum Centre for Advanced Histology and Microscopy. Semi-thin sections (0.5–1.0 μm) of the corpus callosum were collected on glass slides and stained with 1% toluidine blue for region selection. Ultra-thin (0.1 μm) sections were subsequently collected on 3 × 3-mm copper grids. Specimens were examined using a JEOL1011 TEM, and images were collected with MegaView II CCD cooled camera operated with iTEM AnalysSIS software (Olympus Soft Imaging Systems).

### Statistical Analysis

In assessing MBP with or without permeabilization, there were three variables: (1) treatment with cuprizone, (2) permeabilization condition, and (3) collection time (cuprizone or recovery). To adequately preform statistics with three variables, we employed a three-way ANOVA mixed-model analysis and Tukey’s multiple comparisons *post hoc* testing to compare means. In all other cases, two-way ANOVA analysis was performed with Tukey’s multiple comparison *post hoc* testing to compare means. All statistical analyses were performed using GraphPad Prism version 8.1.1 for MacOS, GraphPad Software, San Diego, CA, United States. In all cases, the statistical tests and error are reported in the figure legends.

## Results

### Using SCoRe Imaging to Quantify Cortical Myelin in Fixed Frozen Sections

Spectral confocal reflectance imaging is a powerful, label-free tool to visualize compact myelin *in vivo* (Schain et al., 2014). It has been demonstrated that SCoRe imaging is compatible with fixation (Schain et al., 2014), and it has recently been adopted to assay compact myelin following administration of putative therapeutic agents in animal models of peripheral and central demyelination (Gonsalvez et al., 2017; Govier-Cole et al., 2019). In fixed frozen sections, SCoRe imaging can only be applied when the tissue is sectioned with the myelin internodes and axons parallel to the cut surface of the section (i.e., coronal sections for the corpus callosum; Figure 1). The composition of compact myelin, overlapping layers of lipid-rich sheaths, leads to constructive and destructive interference of wavelengths; such wavelengths are preferentially reflected whereas others are suppressed (Schain et al., 2014). This means single laser lines are preferentially reflected from different components along the length of an internode (Schain et al., 2014; Figures 1B,C). We find that this pattern of reflection is maintained in coronal sections of the corpus callosum (Figure 1C). It is theoretically possible to estimate axon diameter by evaluating the orthogonal SCoRe reflection pattern generated by *z*-stack confocal images. In the corpus callosum, the outer diameter (myelin + axon) corresponds to TEM images taken from the same individuals. However, the reflections from the upper and lower surfaces of myelinated callosal axons are unable to be resolved (Figure 1D), indicating these axons are too small to be measured by this technique. For comparison, a TEM image taken from the same individual animal is shown (Figure 1E).

### Using SCoRe Imaging to Quantify Cortical Myelin Changes Following Demyelination

To test the sensitivity of SCoRe imaging as a quantitative approach to measure changes to compact myelin in the context of demyelination, we employed the cuprizone model. Cuprizone is a copper chelator that leads to oligodendrocyte death and subsequent demyelination. Commencing at 6–8 weeks of age, mice were fed 0.2% cuprizone for 3 weeks (*n* = 3), 4 weeks (*n* = 3), 6 weeks (*n* = 3), 6 weeks with 1-week recovery following cuprizone withdrawal (*n* = 3), and 6 weeks with 3-week recovery following cuprizone withdrawal (*n* = 3). To compare with normal myelination, at each time point, age-matched control animals on normal chow were also collected (*n* = 3/group). SCoRe imaging was highly sensitive to the changes in compact myelin in the corpus callosum that occur because of normal aging, as well those that occur in response to ongoing cuprizone exposure (Figure 2A). Quantifying the SCoRe signal by assessing the percentage of the total cross-sectional area of the corpus callosum in each section positive for SCoRe signal (pixels) revealed that cuprizone resulted in a significant (*p* < 0.01) decrease in SCoRe signal in both rostral (Figure 2B) and caudal (Figure 2C) corpus callosum from 3 weeks of cuprizone administration, indicative of demyelination and disruption to compact myelin integrity. Moreover, SCoRe was able to detect significant differences in the level of myelination between cuprizone and healthy control groups during 1 and 3 weeks of recovery (Figures 2B,C), suggesting that SCoRe is a valid method to measure the extent of myelin integrity during recover. Notably, quantification of SCoRe signal was also sensitive enough to detect a significant increase of compact myelin in both rostral and caudal corpus callosum in the age-matched healthy control mice from ∼10 to ∼14 weeks of age (Figures 2B,C), consistent with published data showing that the murine corpus callosum acquires about 15% of its total myelin over this period (Sturrock, 1980). This finding suggests that SCoRe imaging is sensitive to identify the normal developmental processes of myelination that are ongoing over this period, consistent with previous studies undertaken in the cerebral cortex (Hill et al., 2018; Hughes et al., 2018). Together, our data demonstrate that SCoRe is a sensitive imaging modality that can be used to measure the extent of not only myelin damage/repair in animal models of diseases but also ongoing myelination during normal development.

### Combined Use of SCoRe Imaging With Immunohistochemistry to Identify Areas of Structural Damage

**FIGURE 3:**
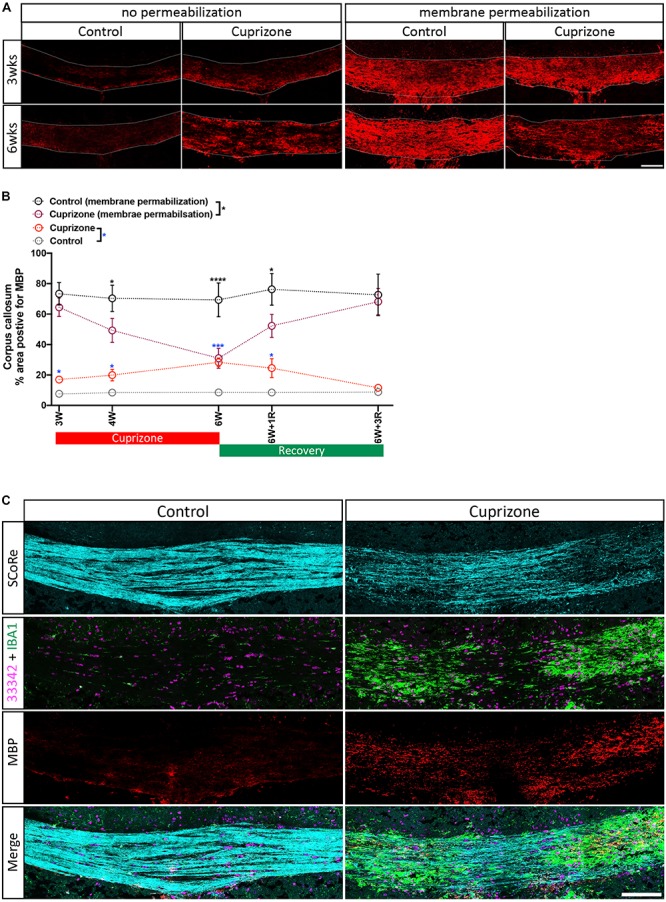
Antigenicity of myelin proteins is increased following myelin damage. **(A)** Representative micrographs of myelin protein MBP immunostaining in the corpus callosum of healthy control and cuprizone-challenged mice without or with membrane permeability steps being employed in the histological processing (scale bar, 100 μm). **(B)** Quantification of the percentage area positive for myelin protein MBP immunoreactivity. Irrespective of treatments, the area of pixels positive for MBP immunofluorescence is greater in staining with membrane permeabilization steps than in those without. When membrane permeabilization is omitted from the tissue processing, the area of pixels positive of MBP immunofluorescence is elevated in cuprizone-fed mice compared with healthy controls (*n* = 3 mice per group, statistics stars indicate a significance between cuprizone and control groups by three-way ANOVA testing using multiple comparisons, ^∗^*P* < 0.05, ^∗∗^*P* < 0.01, ^∗∗∗^*P* < 0.001, ^∗∗∗∗^*P* < 0.0001, data = mean ± SD). **(C)** SCoRe imaging combined with immunofluorescence imaging against MBP and IBA-1 in the corpus callosum of healthy control and cuprizone-challenged mice. Areas with reduced SCoRe signal indicate demyelination, which overlaps with elevated microglia infiltration (IBA-1-positive cells) and enhanced intensity of MBP immunoreactivity (scale bar, 100 μm). MBP, myelin basic protein.

Typical approaches to quantify the level of myelin protein expression by immunohistochemistry (or immunofluorescence) require aggressive membrane permeabilization steps to expose myelin proteins and increase their antigenicity. Using routine membrane permeabilization techniques, we were able to detect changes in the immunofluorescence of MBP over the course of the cuprizone model (Figures 3A,B). However, recent evidence suggests that cuprizone leads to early structural modifications in myelin. Importantly, these structural changes may be required for immune interactions that are likely significant during the course of demyelination (Liu et al., 2010; Caprariello et al., 2018). We hypothesized that the damaged to or loss of compact myelin structure will increase antigenicity and the ability to detect myelin specific proteins in the presence of membrane permeabilization agents/steps. To determine if damaged myelin has a higher level of antigenicity, we performed immunohistochemistry against MBP without processing the tissues with traditional permeabilization steps. We found that after omitting standard tissue permeabilization steps (methanol and Triton X-100), MBP immunofluorescence was almost absent from control mice. This was unsurprising as compact layers of lipid membrane shield myelin proteins, leading to low antigenicity. Surprisingly, we observed a significant increase in MBP immunofluorescence in cuprizone-treated mice compared with controls when membrane permeabilization steps were omitted from the tissue processes. Furthermore, imaging with SCoRe revealed that the immunofluorescence for MBP (in the absence of permeabilization steps) corresponded to the areas of decreased and patchy SCoRe signal, indicative of a loss/damage of compact myelin. No MBP positive staining was detectable in areas where compact myelin is intact, as assessed by SCoRe imaging (Figure 3C). In addition to this, the detection of IBA-1 clearly demonstrates the profound and highly localized infiltration of macrophages to these demyelinated regions, with IBA-1+ cells sparse in regions of intact compact myelin detected by SCoRe (Figure 3C). Overall, our results provide strong evidence that structural breakdown of compact myelin such as that occurring in the cuprizone model results in increased detection of myelin proteins using antibody-based assays when permeabilization steps are not carried out. Furthermore, this approach may present a means to positively identify areas within tissues where myelin structure has been damaged even early on in the cuprizone model.

### SCoRe Imaging Can Be Used to Quantify Atypical Appearing Myelin and Myelin Debris

**FIGURE 4:**
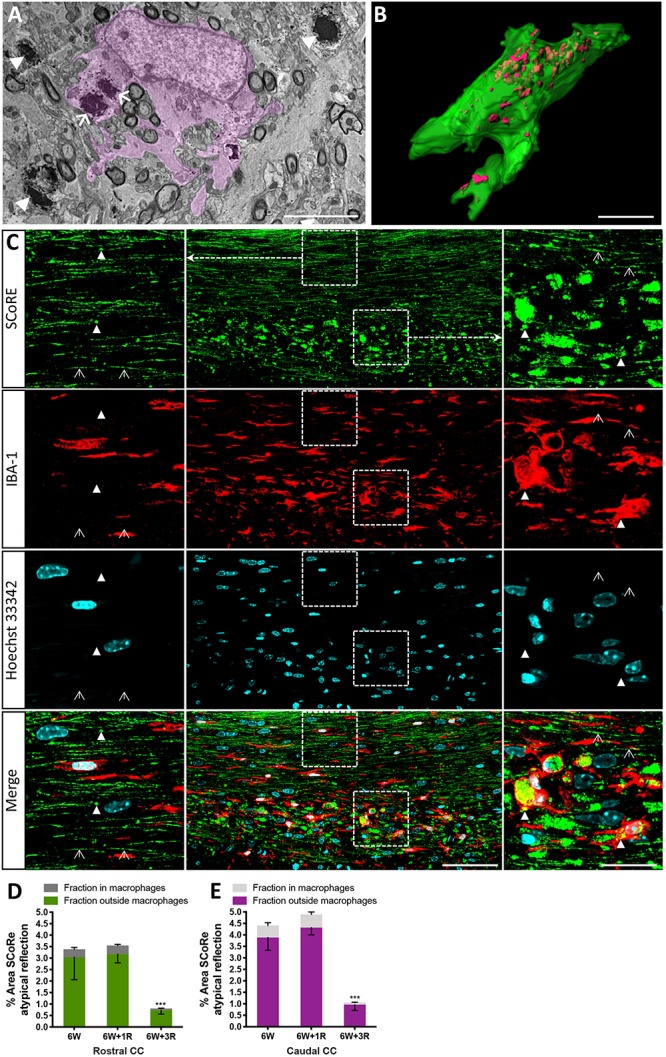
Pathological myelin and myelin debris can be quantified using SCoRe imaging. **(A)** Transmission electron microscopy micrograph through the corpus callosum at the peak of demyelination (after 6 weeks of feeding of 0.2% cuprizone in normal chow). A CNS macrophage (pink shading) containing electron dense myelin debris (arrows). Compact myelin surrounding axons in a **(B)** *Z*-projection and 3D reconstruction of an IBA-1-positive macrophage (green) with myelin debris identified by SCoRe (red) (scale bar, 5 μm). **(C)** Coronal single *Z* plane image through the corpus callosum of a mouse administrated with cuprizone for 6 weeks followed by 1-week recovery. The SCoRe reflectance from normal-appearing compact myelin is identified by open arrowheads. Compact myelin debris is indicated with solid arrowheads and can be observed within IBA-1-positive macrophages (scale bar, 10 μm). **(D,E)** The percentage area of myelin debris in the rostral **(D)** and caudal **(E)** corpus callosum at the peak of demyelination and during recovery. At all points, ∼10% of the debris was identifiable in IBA-1 positive macrophages (statistics: *n* = 3 mice per group for each data point, ANOVA with multiple comparisons, and Tukey’s *post hoc*, ^∗∗∗^*P* < 0.01, data = mean ± SD). SCoRe, spectral confocal reflectance; CNS, central nervous system.

**FIGURE 5:**
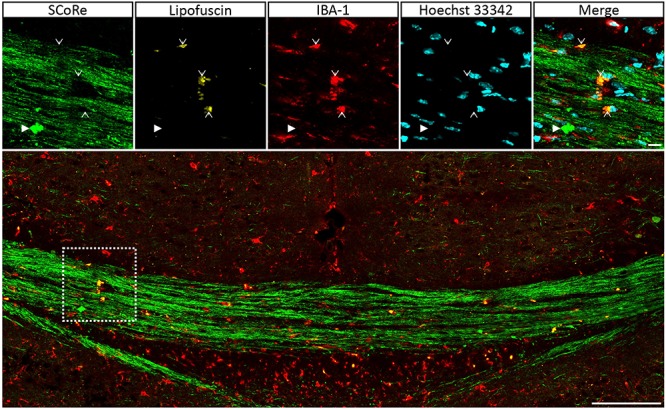
Autofluorescent lipofuscin aggregations in IBA-1 macrophages do not generate a SCoRe signal. Coronal single *Z* plane image through the corpus callosum of a mouse administrated with cuprizone for 6 weeks followed by 3 weeks of recovery. The autofluorescent lipofuscin is localized within IBA-1+ macrophages (open arrowheads) but not colocalized with compact debris (closed arrowhead). Scale bar of 10 μm for the small images and 100 μm for the large images. SCoRe, spectral confocal reflectance.

It is well established that experimentally generated myelin debris, injected back into remyelinating tissues, exerts an inhibitory effect on oligodendrocyte precursor cell (OPC) differentiation and has the capacity to increase macrophage activity (Kotter et al., 2006). At present, the only means to visualize myelin debris is via TEM. Our TEM analysis of callosal tissue following 6 weeks of cuprizone reveals the presence of myelin debris, both within and outside of macrophages (Figure 4A). Importantly, the imaging demonstrates that debris artifacts are densely compacted yet structurally distinct from those of normal white matter (Figure 4A). Unfortunately, TEM is extremely time-consuming and only samples a tiny fraction of tissue, such that quantification of myelin and debris in this way is likely not completely representative and unreliable. Previous studies have indicated that myelin debris can be identified using SCoRe on the basis of its differential reflection pattern (Hill et al., 2018). To determine the extent of myelin debris and whether it has been engulfed by macrophages after 6 weeks of cuprizone exposure (typically considered peak demyelination) and whether it changed after 1 and 3 weeks of remyelination, combined IBA-1 immunofluorescence and SCoRe images were analyzed (Figures 4B,C). This revealed that there was no change in the percentage area positive for myelin debris in either the rostral (Figure 4D) or caudal (Figure 4E) corpus callosum of mice fed cuprizone for 6 weeks than of those allowed to also remyelinate for 1 week. Intriguingly, there was also no change in the percent of myelin debris localized within IBA-1+ macrophages between peak demyelination and 1-week remyelination (Figures 4D,E). At 3 weeks of remyelination, there was a profound decrease in profiles that resembled myelin debris by SCoRe imaging (Figure 4E). However, during the remyelinating process, approximately 10% of all detected myelin debris was found within macrophages (Figure 4E), indicating that, at any instant, ∼90% of myelin debris detectable by SCoRe imaging is observed outside of macrophages and during remyelination following cuprizone. Furthermore, to investigate if SCoRe signal captures compact myelin or not a unique myelin composition of lipid, we performed SCoRe imaging together with lipofuscin as well as IBA-1. Lipofuscin is a lipid containing residue and is also autofluorescent. We found that at 3 weeks of remyelination, autofluorescent lipofuscin aggregates were observed within IBA-1+ macrophages (Figure 5). Although these aggregations are lipid, they are not compact and hence not colocalized with the SCoRe signal (Figure 5), further demonstrating that the laser light can only be reflected of unique compacted myelin, rather than the membrane composition of lipid. Together, we show that combining SCoRe imaging with immunofluorescence is an exciting way to detect and quantify myelin debris *ex vivo.*

## Discussion

Quantification of the extent of myelin damage and repair is highly dependent upon techniques being used. This study is the first to quantitatively analyze myelin integrity after a demyelinating insult *in vivo* has used SCoRe microscopy, a novel label-free structural-based myelin imaging technique. We have demonstrated that this technique enables high-throughput quantitative analysis of myelin integrity of the whole corpus callosum in a murine model of CNS demyelination, avoiding the confounding changes to myelin protein antigenicity that occur as a result of myelin injury and limited sampling capacity of TEM. Through combining SCoRe imaging with immunohistochemistry, we have quantified the amount of myelin debris and found the majority of myelin debris is localized outside of macrophages even during remyelination. In summary, we demonstrated that SCoRe is an effective and high-throughput tool to quantify changes in compact myelin structure owing to demyelination *in vivo*, implicating its broad applications in other model systems where changes in myelination are of interest.

### SCoRe Enables High-Throughput Quantitative Analysis of Myelination After Injury *in viv*o

Currently, relatively few approaches have been used for *in vivo* imaging of myelinated axons and myelin-forming glial cells. For example, confocal and two-photon microscopy has been used with genetically labeled fluorescent markers to image oligodendrocytes and myelin sheaths within zebrafish (Kirby et al., 2006; Czopka et al., 2013; Simons and Lyons, 2013; Hines et al., 2015; Baraban et al., 2018). However, imaging myelinated axons or compact myelin sheath using fluorescent reporters requires complex imaging systems, and labeling myelin sheaths with fluorescent dyes is often inconsistent, creating challenges for quantitative analysis of myelin dynamics over time in rodent disease models *in vivo*. SCoRe uses a conventional confocal microscope, making it easy to implement. Other label-free imaging modalities could also be applied to image myelin and myelin debris. Coherent anti-Stokes Raman scattering (CARS) microscopy utilizes intrinsic molecular vibration of lipid structures to enable imaging of myelinated fibers (Wang et al., 2005) and *in vivo* mouse brain (Fu et al., 2008). However, these imaging techniques require complex setups, and the equipment required for this complex imaging is rarely available, making them difficult to implement and hence not widely used (Farrar et al., 2011), whereas SCoRe imaging requires confocal microscopy, which is commonly available, easy to implement, and readily accessible. Importantly, those imaging techniques that enable label-free quantification of myelin that is second or third harmonic generation (S/THG) depends on the molecular composition of the myelin for the generation of its signal and requires the use of multiphoton-specialized equipment that may not be readily available. The key point of difference between the signals collected using CARS and S/THG compared with SCoRe is that the SCoRe signal relies on the structural organization of the lipid rather than the molecular structure of the lipid *per se*. Thus, SCoRe allows the imaging of the unique compact myelin than do other label-free imaging techniques. This is highly important, as the SCoRE signal is binary only to compact myelin that reflects the incident light and the compacted lipid layers that are unique to myelin (Schain et al., 2014; Hill et al., 2018). In this study, we applied SCoRe imaging directly onto frozen brain sections of adult mice and have demonstrated its capacity to be combined with immunohistochemistry. Consistent with the previous study (Schain et al., 2014), we found that SCoRe is highly sensitive to myelin changes during normal aging and demyelination and allows repeated imaging over a large anatomical ROI with high resolution. Importantly, the acquisition of SCoRe signals enables both qualitative and quantitative analyses of changes to compact myelin during cuprizone-induced CNS demyelination.

Several studies have reported that cuprizone induces CNS demyelination in anatomically diverse regions, including the cerebral cortex (Gudi et al., 2009; Skripuletz et al., 2011), cerebellum (Groebe et al., 2009), hippocampus (Norkute et al., 2009), and the key white matter tract corpus callosum (Gudi et al., 2009; Skripuletz et al., 2011), with the corpus callosum recognized as the most prominent and reproducible demyelinating region (Skripuletz et al., 2011). TEM analysis of myelin ultrastructure suggests that, in the murine cuprizone model, there exists a rostral–caudal pattern of myelin damage in the corpus callosum (Stidworthy et al., 2003; Steelman et al., 2012), with the caudal part exhibiting a greater and more reproducible demyelination (Steelman et al., 2012), whereas the extent of demyelination and remyelination is more variable in the rostral portion (Stidworthy et al., 2003). Indeed, a limited number of studies conclude that the rostral corpus callosum displays subtle demyelination in the cuprizone model (Stidworthy et al., 2003; Steelman et al., 2012) and that analysis using traditional histology or TEM fails to reveal a significant difference between cuprizone-challenged mice and unchallenged controls (Stidworthy et al., 2003; Steelman et al., 2012). Our study demonstrates that SCoRe has the sensitivity required to quantify the extent of myelin damage in both rostral and caudal corpus callosum during cuprizone-induced demyelination, identifying significant differences between cuprizone-challenged mice and age-matched control mice. Collectively, the literature together with our data suggests that although the EM technique is an excellent approach to quantify myelin, it does not have the sampling power to account for the innate myelin variability in the rostral corpus callosum. In this study, SCoRe microscopy markedly facilitates sampling of large areas of tissues, allowing high-throughput and sensitive detection of myelin damage/loss *in vivo*. In addition, corpus callosum undergoes ongoing myelination over the life span in mice (Sturrock, 1980). Our study also identifies the temporal change of myelination in the corpus callosum of mice during late development and early adulthood, which is consistent with the age-dependent increase previously reported using traditional TEM analysis (Sturrock, 1980). Together, our findings highlight the advantage of adopting SCoRe to quantitatively assess myelin integrity over a large anatomical ROI *in vivo*.

### ScoRe Is Independent of Myelin Protein Antigenicity After Myelin Injury *in vivo*

Analysis of myelin damage and repair after an injury is more complex than normal development owing to the presence of damaged myelin sheaths in lesions. In addition to TEM, immunohistochemical analysis of myelin protein expression is a commonly used conventional approach to assess myelination after injury *in vivo*. Indeed, many publications that have assessed demyelination and remyelination via this technique utilize some forms of antigen retrieval to improve the antigenicity of proteins located between the lamellae of compact myelin (Lindner et al., 2008; Gudi et al., 2009; Xu et al., 2009). In the context of myelin injury such as the cuprizone model, damage to myelin sheaths increases the exposure of internal myelin proteins, resulting in greater antibody recognition and is consequently not a true selective representation of myelinated axons. In this study, we found that immunohistochemical staining against the myelin protein MBP in cuprizone-demyelinated brain without using detergent or antigen retrieval revealed counterintuitive results with a significantly higher level of myelin protein expression in the corpus callosum after cuprizone-induced demyelination. Importantly, comparison with the SCoRe signal was highly informative, as the high level of MBP immunofluorescence corresponded to decreased SCoRe signal. This indicates immunostaining for myelin proteins without membrane permeabilization may be a good surrogate for quantifying the extent of myelin damage, rather than the level of intact myelin.

Use of immunostaining for myelin proteins as a surrogate marker for myelin damage is the inverse of how myelin protein immunofluorescence is traditionally used. It also highlights the variable nature of myelin immunostaining, which depends on the extent of physical disruption by either damage or membrane permeabilization methods. Although this problem of structural disruption and antigenicity may be overcome with antigen retrieval steps to a certain extent, this is not always the case. Xu et al. (2009) reported that the use of detergent during immunostaining of myelin proteins resulted in high fluorescent intensity of the myelin protein MBP in the corpus callosum at the peak of demyelination of cuprizone-fed mice compared with unchallenged control mice. Hence, the amount of myelin protein assessed via immunohistochemistry failed to accurately identify level of intact myelin after injury. This represents a significant limitation for the use of fluorescent intensity to quantify myelin. SCoRe microscopy exploits the reflective property of compact myelin (Schain et al., 2014; Hill et al., 2018; Hughes et al., 2018), enabling direct visualization of the structure of compact myelin and objective quantitative analysis of myelin integrity through postimage acquisition. It allows an antibody-free imaging modality, which is not compatible with membrane permeabilization methods during immunohistochemical staining. Moreover, immunostaining against myelin proteins does not discriminate intact compact myelin from disorganized atypical myelin such as myelin debris, ultimately leading to an overestimation of myelination, confounding the analysis of myelin integrity (Skripuletz et al., 2011). In a study by Lampron et al. (2015), higher levels of MBP fluorescent intensity, initially thought to indicate greater myelination, were, in fact, abnormal patterns of myelin. Concordant with this finding, in our study, we have demonstrated that myelin protein expression detected by immunohistochemistry is found in areas where there is a low level of compact myelin signal via SCoRe, accompanied via a high density of macrophages. Therefore, our study together with others suggests that immunohistochemical analysis of myelin protein expression is not an accurate measurement of compact myelin after injury and that ScoRe has a unique capacity to measure intact myelin *in vivo*.

### SCoRe Imaging Enables Quantitative Analysis of Myelin Debris After Injury *in vivo*

Pathology from postmortem MS brains indicates that myelin debris exists in MS lesions as clustered aggregations of compact myelin fragments (Prineas et al., 1984). Myelin debris is present within resident and infiltrating peripheral macrophages, indicative of debris clearance by both microglia and macrophages (Prineas et al., 1984). Currently, beyond *in vivo* imaging with appropriate fluorescent reporters, TEM is the most accessible method to detect myelin debris. However, the chance of encountering a debris event in a TEM image is low owing to the extremely small size of sample regions, limiting its utility for accurately quantitating the amount of myelin debris. Using the SCoRe imaging, Hill et al. (2018) identified not only the ongoing cortical myelination but also occasional debris fragments colocalized in IBA-1-positive microglia in mice beyond 24 months of age *in vivo*. Consistent with the published work, we show that SCoRe microscopy enables visualization of myelin debris with a distinct appearance from healthy intact compact myelin on *ex vivo* slides. Importantly, we, for the first time, demonstrate that SCoRe microscopy allows quantification of intracellular and extracellular myelin debris. Our data have shown that cuprizone-challenged animals display a substantially higher amount of myelin debris in the corpus callosum than have age-matched healthy control mice. Interestingly, we found only approximately 10% of myelin debris resides within microglia irrespective of callosal regions, whereas the vast majority (90%) is located outside of cells without being in intact axons. There has been continuous interest in the association between the presence and clearance of myelin debris and remyelinating efficiency. Several studies have revealed that efficient removal of myelin debris by microglia is a prerequisite for facilitating remyelination (Kotter et al., 2005; Lampron et al., 2015). Furthermore, a delay in myelin debris clearance has been shown to obstruct the differentiation of OPCs into mature myelin-forming cells (Kotter et al., 2005), hindering myelin repair (Lampron et al., 2015). Our study indicates that SCoRe imaging enables quantitative analysis of intracellular and extracellular myelin debris after injury *in vivo*, indicating that this is a new and powerful approach in evaluating the therapeutic efficacy of new myelin repair strategies that may incorporate efficient myelin debris clearance.

This study demonstrates an application of SCoRe imaging for assessing myelin integrity, overcoming the confounding myelin protein antigenicity after injury and limited sampling capacity of TEM. Despite these advantages, current SCoRe imaging also has limitations. It is important to note that the compact myelin has to be in a specific orientation to the incident light in order generate the SCoRe signal (Schain et al., 2014). In combination with immunohistochemistry, SCoRe imaging is able to capture mature myelinating oligodendrocytes and the longitudinal growth of myelin sheaths, allowing the measurement of the number and length of myelin internodes per oligodendrocyte (Hughes et al., 2018). However, the light reflective nature of this imaging method for assessing myelin does not equip it to determine the radial growth of myelin sheaths such as myelin membrane thickness, an important indicator of myelin development and regeneration. Given this orientation limitation, the quantification of compact myelin debris assessed via SCoRe imaging would be underestimated.

## Conclusion

In summary, we have demonstrated that SCoRe microscopy is a high-throughput imaging modality that can sensitively detect and quantify changes in myelin integrity including compact myelin and myelin debris. It supplements TEM for more defined analysis of myelin integrity. Being able to detect myelin debris, this label-free and easy-to-implement technique can replace the conventional approach of immunohistochemistry against myelin proteins and supplement TEM regarding sampling size. Application of SCoRe imaging in future preclinical analysis of putative MS therapies will allow efficient and accurate assessment of drug efficacy in promoting myelin repair.

## Data Availability Statement

All datasets generated for this study are included in the article/supplementary material.

## Ethics Statement

The animal study was reviewed and approved by The Florey Institute for Neuroscience and Mental Health Animal Ethics Committee of the University of Melbourne (Australia).

## Author Contributions

DG and JX conceived the study. DG, SY, RW, and GC performed the experiments. DG and SY analyzed the data. DG, SY, and JX wrote the manuscript. DG, JF, and JX reviewed and revised the manuscript. All authors read, revised, and approved the submitted version of the manuscript.

## Conflict of Interest

The authors declare that the research was conducted in the absence of any commercial or financial relationships that could be construed as a potential conflict of interest.
